# A human-in-the-loop approach for enhancing mobile robot navigation in presence of obstacles not detected by the sensory set

**DOI:** 10.3389/frobt.2022.909971

**Published:** 2022-11-29

**Authors:** Francesco Ferracuti, Alessandro Freddi, Sabrina Iarlori, Andrea Monteriù, Karameldeen Ibrahim Mohamed Omer, Camillo Porcaro

**Affiliations:** ^1^ Department of Information Engineering, Università Politecnica delle Marche, Ancona, Italy; ^2^ Department of Neuroscience and Padova Neuroscience Center (PNC), University of Padova, Padova, Italy; ^3^ Institute of Cognitive Sciences and Technologies (ISCT)-National Research Council (CNR), Rome, Italy; ^4^ Centre for Human Brain Health, School of Psychology, University of Birmingham, Birmingham, United Kingdom

**Keywords:** human-in-the-loop approach, robot navigation, obstacle avoidance, EEG signal, BCI

## Abstract

Human-in-the-loop approaches can greatly enhance the human–robot interaction by making the user an active part of the control loop, who can provide a feedback to the robot in order to augment its capabilities. Such feedback becomes even more important in all those situations where safety is of utmost concern, such as in assistive robotics. This study aims to realize a human-in-the-loop approach, where the human can provide a feedback to a specific robot, namely, a smart wheelchair, to augment its artificial sensory set, extending and improving its capabilities to detect and avoid obstacles. The feedback is provided by both a keyboard and a brain–computer interface: with this scope, the work has also included a protocol design phase to elicit and evoke human brain event–related potentials. The whole architecture has been validated within a simulated robotic environment, with electroencephalography signals acquired from different test subjects.

## 1 Introduction

An emerging requirement in the human–robot interaction (HRI) is that of effectively using the feedback from the human operator to modify the robot behavior. In cooperative tasks, such feedback allows indeed to handle factors which may negatively affect the cooperative performance and possibly mitigate their effects as investigated in the literature (Iturrate et al., 2010; Iturrate et al., 2012; Zhang et al., 2015; Salazar-Gomez et al., 2017). Human feedback becomes even more important in all those situations where it can increase safety. Among the robotic fields in which safety is of utmost concern, the assistive one plays an important role: in assistive robots, the interaction between the robot and user is close and recurrent, and the user relies on the robot to achieve tasks that he/she could not perform on his/her own. This is especially the case of physically assistive robots, which provide mobility to the user, such as smart wheelchairs or walkers, which are specialized types of mobile robots, whose main goal is to reach a target safely and accurately together with the user (Aljalal et al., 2020). In order to reach the target, the mobile robot relies on a navigation stack which exploits available maps and the information provided by proprioceptive and exteroceptive sensors (Patle et al., 2019), required at a local level to avoid obstacles. Common errors during navigation are indeed represented by unexpected environmental conditions, algorithmic errors, or wrong sensor readings, such as in the case of “negative obstacles”, namely, obstacles represented by holes in the ground or regions that lay below the ground surface (Herghelegiu et al., 2017). In order to enhance the HRI, the user can become an active part of the control loop by providing a feedback to the robot in order to augment its artificial sensory set, thus extending its capabilities to detect specific obstacles.

There are several devices which can be used to provide feedback to a robotic platform. Especially in the last years, HRI supported by the brain–computer interface (BCI) has emerged as a research topic to allow the user to supervise different robot tasks providing direct feedback (Millán et al., 2010), (Krishnan et al., 2016; Rashid et al., 2020; Mridha et al., 2021). An EEG-based robot control has been addressed in several works in the literature (Tariq et al., 2018; Kim et al., 2020), including mobile robots, according to the so-called shared control paradigm (Aljalal et al., 2020). Most of the time, the mobile robot is directly controlled *via* the BCI, such as in Choi (2012), where a smart wheelchair is proposed that turns to the left, to the right, or moves forward according to what the user imagines. This approach is also used to directly navigate the wheelchair around an obstacle, but without interaction with the robotic navigation system. In order to reduce the control effort from the user side, the brain-controlled mobile robot can also exploit part of the autonomous navigation capabilities. In Satti et al. (2011), the user controls the robot by the BCI, and the autonomous navigation is triggered only in the situations of obstacle avoidance and corridor following. Nevertheless, only a few works do address the problem of augmenting the obstacle avoidance capabilities of a mobile robot *via* the BCI (Bi et al., 2013). In Tonin et al. (2010), a service robot is presented, whose default behavior is to move forward at a constant speed, and upon the reception of a mental command, it turns left or right by 30°. To avoid obstacles, the robot employs infrared sensors and applies one of two policies, namely, to use the BCI for direct navigation or demand the obstacle avoidance task to the navigation module, thus not being robust against negative obstacles. To address this problem, Puanhvuan et al. (2017) proposed a brain-controlled wheelchair (BCW) with an advanced obstacle sensing technology, namely, a main laser scanner for long distances together with a rotating laser scanner for short distances in 3D. This solution has, however, increased costs (with respect to the commonly used 2D laser scanners) and does not work with transparent objects, such as glass, acrylic, and clear plastics.

To the best of the authors’ knowledge, the problem of obstacle avoidance *via* EEG signals during navigation of a mobile robot is thus still open (Lopes-Dias et al., 2019) and remains a challenging one (Rashid et al., 2020). Indeed, the proposed paper would contribute to the literature by proposing a method to integrate the navigation stack of a mobile robot with an external trigger, in order to deal with obstacles which are not correctly detected by the mobile robot. More in detail, this work contributes to the literature by proposing

• a human-in-the-loop approach, where the human can provide a feedback to the robot in order to augment its artificial sensory set and extend its capabilities to detect specific obstacles, such as negative obstacles (e.g., a hole in the ground);
• a method to update the robot navigation stack, commonly used in robotic navigation, when the human feedback is received, in order to avoid obstacles detected in such a way;
• a preliminary way to generate human feedback by means of a BCI, based on a protocol to elicit the event-related potentials (ERPs) when an obstacle, invisible to the robot sensory set, and thus not detected, is instead sensed by the human operator (Salazar-Gomez et al., 2017).

More in detail, the mobile robot object of the study is a smart wheelchair, which can navigate indoors, while avoiding possible obstacles without any human intervention, by using only the available sensors and intelligence mounted on board. A feedback from the human operator is triggered whenever he/she senses an obstacle which is not detected by the robot sensory set. The robot navigation stack is then modified in order to receive human feedback and generate a virtual obstacle, the effect of which is to change the local trajectory planner of the robot, in order to avoid the obstacle. Then, a BCI is introduced as a feedback interface, and the related protocol to elicit the ERP signal in the presence of obstacles is presented. The overall architecture has been first tested by using a keyboard to generate the feedback, with the aim to validate the baseline performances. Then, the performances achieved with the BCI classifier are presented.

The paper is structured as follows: Section 2 presents materials and methods, introducing the proposed approach for the human-in-the-loop solution, with the description of the hardware and software implemented and the simulation environment. In detail, the section describes how path planning and obstacle detection have been implemented together with the realized BCI protocol and the EEG signal pre-processing. In Section 3, the authors present the experimental results with respect to the proposed task about the robot navigation around and through the obstacles and the ERP classification. Finally, Section 4 provides a discussion and conclusion about the study.

## 2 Materials and methods

In this section, the authors present the proposed human-in-the-loop approach, implemented in the simulation environment, describing the path planning and the obstacle detection, which is realized through the sensors equipped with the smart wheelchair and supported by the user estimation of the position of the obstacle, detected or undetected by the onboard sensors. Moreover, the BCI protocol, designed to generate the BCI feedback, is introduced. Finally, the integration of such feedback into the ROS framework to change the path planning and avoid the obstacle is presented.

### 2.1 The proposed human-in-the-loop approach

The proposed approach aims to engage the user in the robot navigation loop. First, the approach proposes how to use the human feedback to augment the robot artificial sensory set and extend its capabilities to detect specific obstacles. In order to use this feedback for improving the robot navigation, it is important that the user can provide an estimation of the position of possible undetected obstacles in an effective way. Then, such feedback is used to create a virtual obstacle within the virtual representation built by the robot through its sensory set. In this way, the local trajectory planner of the robot, which cannot distinguish between the real and virtual obstacle, modifies the robot trajectory to avoid it: this approach is effective as long as the position of the virtual obstacle is close to that of the real one. Finally, the feedback is physically generated through a BCI system interface, and a designed protocol that can evoke ERPs is presented.

For the proposed approach to work, it is important that the user can provide an estimation of the position of a real obstacle for the generation of a consistent virtual obstacle, and obstacles not seen by the robot sensory set but detected by the user are perceived as “errors.” As such, the following three tasks were defined, where the first two are focused on robot navigation and the last one is focused on ERP detection.

1 The first task involves the user watching the screen where the smart wheelchair navigates around 10 holes placed in a virtual environment representing a laboratory, namely, along the corridor, in a passage forward, from the laboratory door to the end of the corridor, and backward, for five times in total. The user was asked to press a keyboard key whenever he/she recognized the presence of a hole.
2 The second task involves the user watching the mobile robot that randomly crosses the obstacles from different starting points around the hole for 32 times. Also, in this case, the user was asked to press a keyboard key whenever the obstacle was identified.
3 The third task involves the user being asked to equip the BCI system and press a keyboard key whenever he/she perceives that the wheelchair is passing through a hole or is avoiding the obstacle. The recorded EEG signals were expected to show slow brain waves, either error-related potentials (ErrPs) or in general event-related potentials (ERPs) as a cognitive response of the human brain when observing the robot in a risk situation (Qin and Han, 2009). These signals could be exploited as triggers for the navigation system.

The first two tasks were designed to evaluate the accuracy in estimating the positions of the real obstacles. The estimated coordinates are indeed used to place virtual obstacles on the virtual map reconstructed by the robot sensory set and modify its trajectory accordingly. As a consequence, the error between the actual and estimated obstacle positions must be limited in order for the proposed approach to work. The human feedback can be generated with any common input device (e.g., keyboard, joystick, sip-and-puff, and touchscreen): the addition of the third task permits validating the feasibility of the proposed human-in-the-loop approach when a BCI is adopted as an input device as well. The third task was thus realized for training the classifier of the BCI system interface in order to recognize the ERP as a trigger to the adopted framework, where the errors are related to the presence of undetected obstacles on the robot path.

**FIGURE 1:**
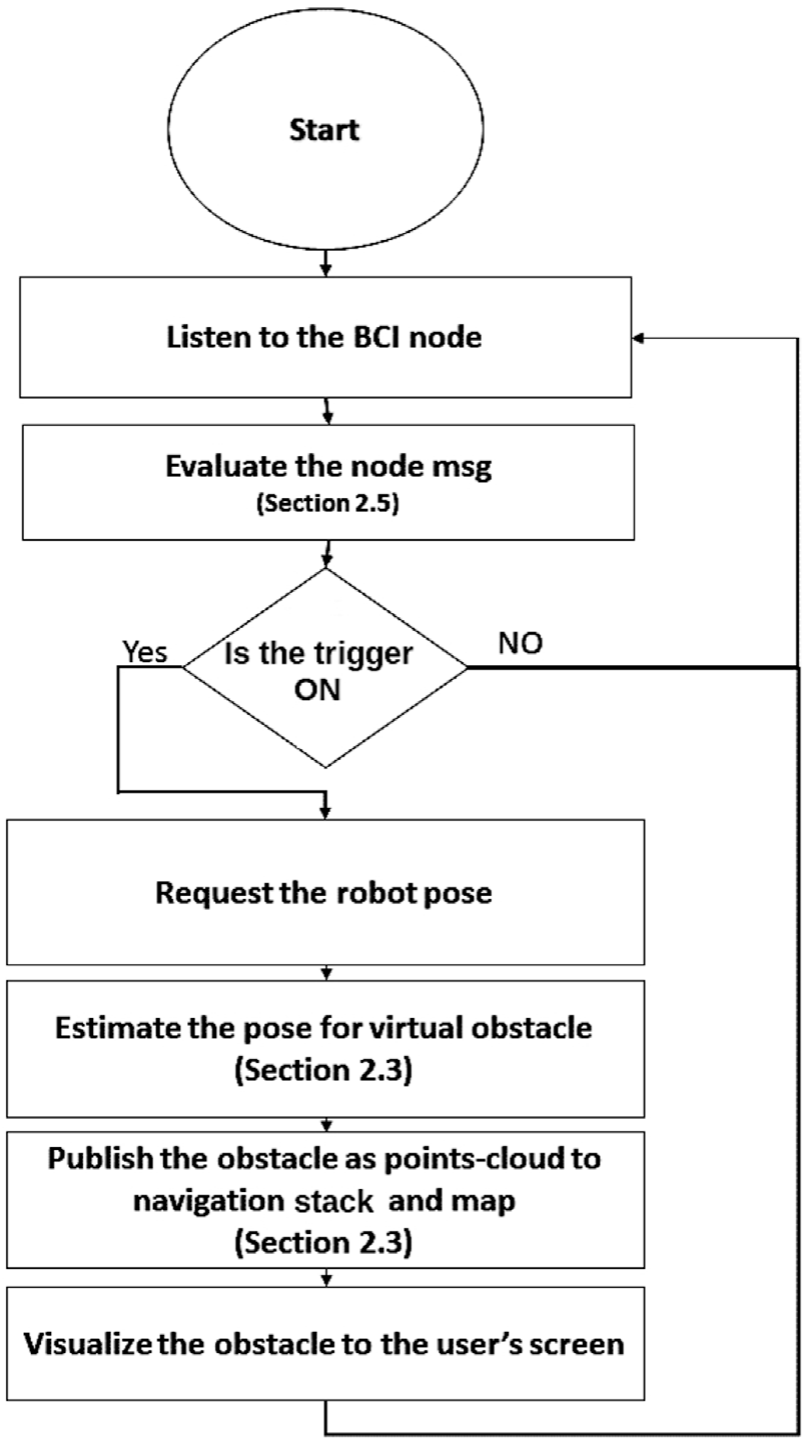
Flowchart of the implemented algorithm from receiving a trigger to the obstacles’ coordinate estimation and ending with new path-planning.

From a software point of view, the EEG signal has been processed in the MATLAB environment and the detected trigger sent to an ROS (robotic operation system) node, specifically created to publish an ROS topic through the Simulink MATLAB–ROS toolbox that produces a Boolean trigger value (i.e., 1 or 0) based on the classification of raw EEG data. Then, the cloud connection was established between MATLAB and ROS. At the same time, in the ROS environment (Quigley et al., 2009), a different node was created that receives the trigger (from the BCI or another input device such as a keyboard) and requests the robot pose in order to eventually create a virtual representation of the obstacle within 2 m on the map. This information was sent to an ROS package designed for robot navigation that manages the wheelchair approach to the obstacle, reducing the velocity and changing the path planning: obstacle avoidance can then be achieved as long as the position of the real obstacle is close to that of the virtual one. The overall flowchart is described in Figure 1.

### 2.2 Hardware, software, and simulation environment description

Environmental data acquired for the robot navigation and path planning are managed by the ROS packages. The proposed algorithms and the methodologies have been developed and improved through different works (Ciabattoni et al., 2019; Ciabattoni et al., 2021; Ferracuti et al., 2021), and experiments were all conducted in simulation to test the communication among the different algorithms and the involved systems.

The ROS includes all packages for robot control and autonomous navigation. The simulated tests have been realized in Gazebo, where a virtual environment for the simulation of the smart wheelchair movement and the acquisition of all the sensors has been recreated. Gazebo is a 3D simulator developed by Open-Source Robotics Foundation by which it is possible to create a 3D scenario with robot obstacles and many other objects. In Gazebo, it is possible to configure the robot as links and joints, and all the equipped sensors are virtualized in order to be used by ROS packages and nodes. It also uses a physical engine for illumination, gravity, inertia, etc. Gazebo was designed to evaluate algorithms for many applications; in fact, it is essential to test the developed robot applications, like error handling, battery life, localization, navigation, and grasping [see Freddi et al. (2021)].

**FIGURE 2:**
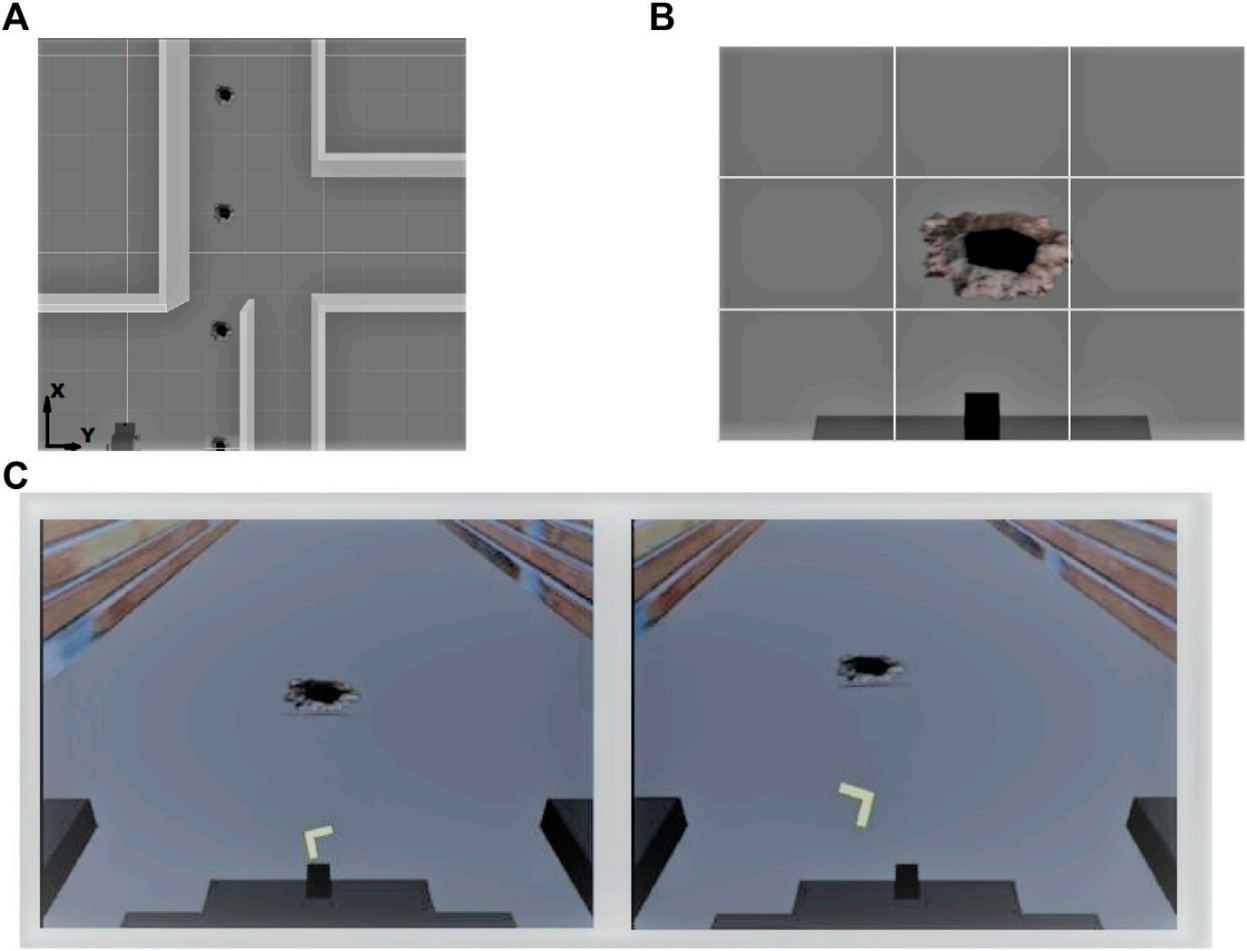
Virtual simulation environment. **(A)** Top-view: the holes are placed along the corridor, separated by 3 m each. **(B)** First-person view: the hole appears in the center of the view when the user gets close to it. **(C)** Two possible visualizations of the directional cues to advise left or right turning task, as visualized in the video streaming in the first-person view as detailed in Section 2.4.

The mobile robot is able to navigate autonomously acquiring environmental data for path planning, by means of external sensors such as the Hokuyo URG-04LX Laser Rangefinder, IMU unit, and two cameras: one camera for localization through QR codes that are positioned on the roof and a second camera that points in front of the robot with an orientation of 30° downward in order to have a camera view of the environment within 2 m, far from the mobile robot. The smart wheelchair navigates in a 3D reconstruction of the Information Engineering Department corridor at Università Politecnica delle Marche. The proposed simulated task consists in navigating in the 3D corridor of the department, avoiding or passing through different holes positioned in the floor that cannot be detected by the sensory set of the robot. The corridor reconstruction was modified by creating 10 holes in the walkway, with a diameter of 20 cm each and separated by 3 m each, as illustrated in Figure 2. The user perspective view during real-time navigation is illustrated in Figure 2.

**FIGURE 3:**
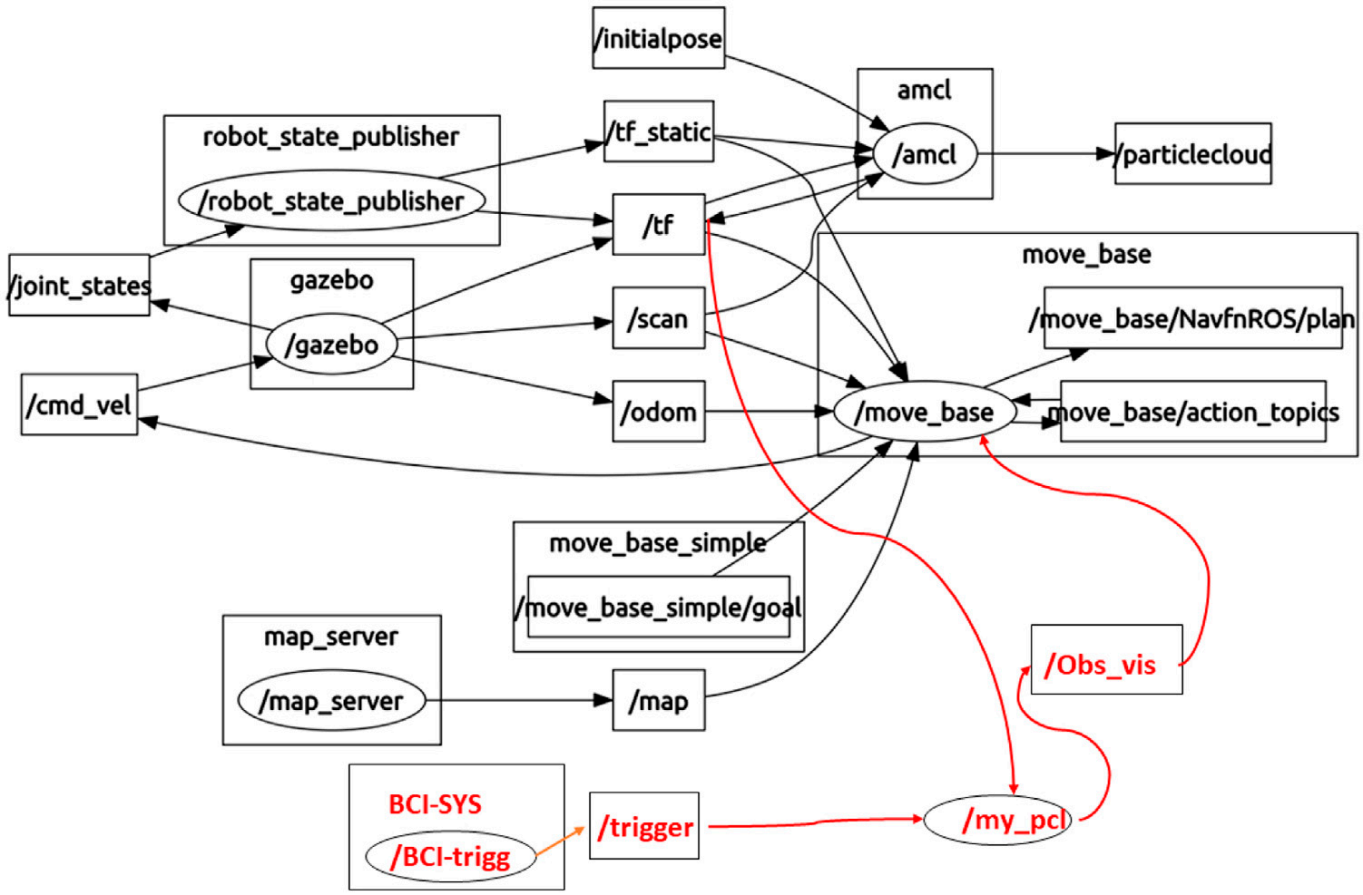
ROS packages and nodes that communicate through topics to exchange information.

The ROS package, dedicated to visualize the robot sensors and state, is Rviz, which also provides a user interface to give robot commands and to monitor the robot, while it is realizing different tasks. Figure 3 shows the ROS structure of the different robot nodes and topics created to record and share the data in the proposed approach. The red connection represents the human feedback flow from the BCI system to the move_based package, which is responsible for the path planning modifications. It should be noted that different triggers (e.g., from a keyboard) can be similarly passed to move_based by adjusting the node specific to the input device.

The monitor used during the training acquisitions with the BCI system had a 38.1 cm diagonal screen and was positioned in a comfortable visual perspective for the user where he/she can see what happens in front of the camera view of the smart wheelchair.

### 2.3 Path planning and obstacle detection

**FIGURE 4:**
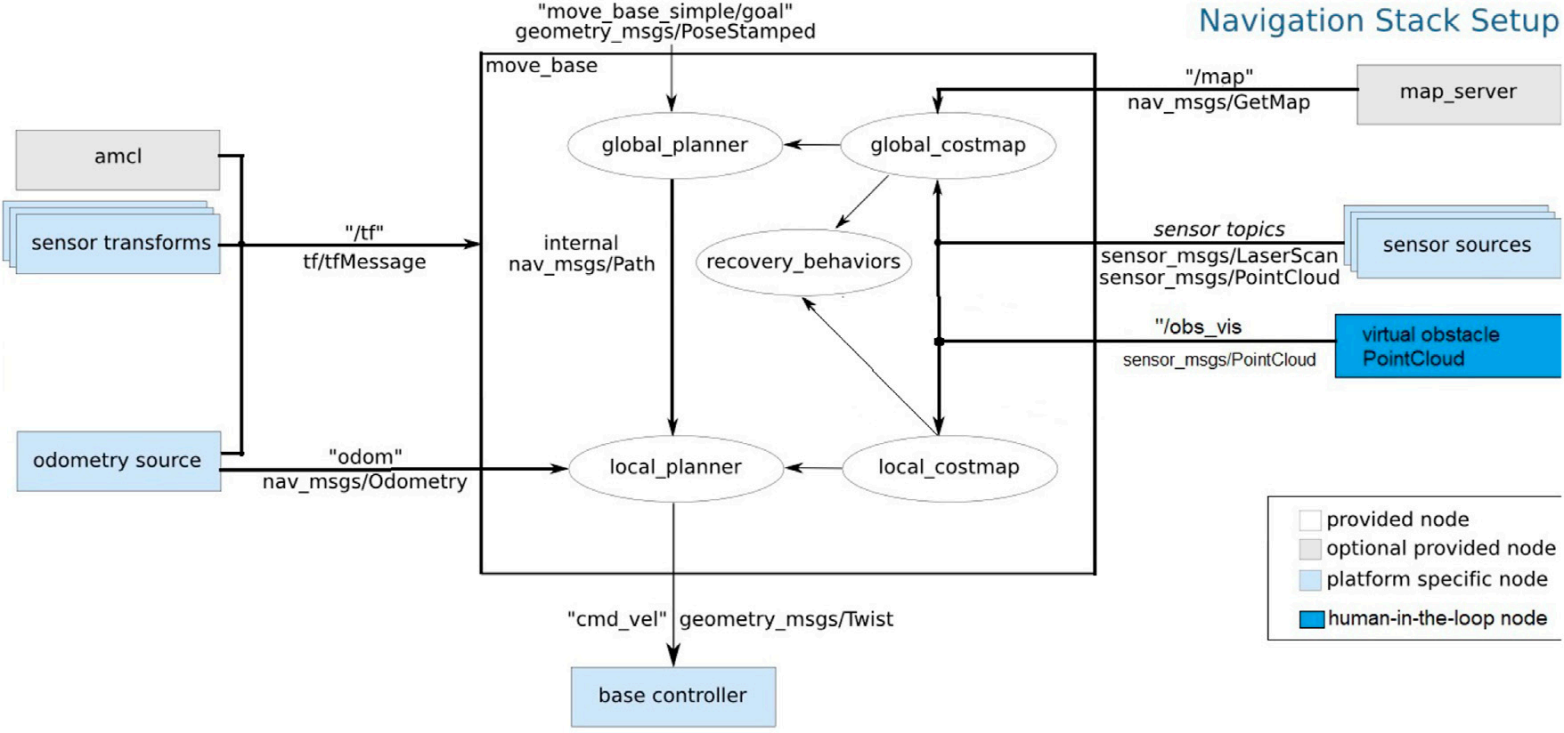
ROS mobile-based navigation stack node with the trigger source [modified from ROS.org (2018)]: the virtual obstacle PointCloud node publishes a topic which has a sensor message for the robot cost map.

In the ROS, it is possible to modify the robot’s path planning at any time by fusing the sensor’s data. With this aim, an ROS structure is developed allowing working at the level of local path planning in order to update the local map when an obstacle is detected. So, in order to communicate the presence of any detected obstacles to the planner (as described in Figure 4), it is necessary to send information to the local planner. Authors have designed a node that estimates the obstacle coordinate on the map, representing it as a virtual object and converting it into a point cloud. The node that publishes the estimated obstacle coordinates requires two pieces of information: the robot position by listening, all the time, to the odometry data and the received trigger message that has been published through ROS topic with the name/trigger. Once the trigger is true (i.e., 1), the node immediately retrieves the current robot pose and extracts the *X* and *Y* coordinates, as well as the orientation angle *θ*, and estimates the obstacle position (based on the nature of the trigger event and robot pose) within 2 m. The estimated coordinates of the hole are calculated based on the robot pose on the map through Eqs 1, 2:

$$X.holes=X.robot+S\times cos\left(\theta \right),$$ (1)

$$Y.holes=Y.robot+S\times sin\left(\theta \right),$$ (2)

where *X*. *robot*, *Y*. *robot*, and *θ* are obtained from listening to the robot pose, which returns in form of quaternions (Cooke et al., 1994), and *S* refers to the hole distance since the camera has been oriented to be centered exactly 2 m away in the space in front of the wheelchair. The *θ* value is calculated as in Eqs 3, 4:

$$\theta \;=2\cdot atan2\left(\sqrt{q_{i}^{2}+q_{j}^{2}+q_{k}^{2}},q_{r}\right),$$ (3)

$$q=q_{r}+q_{i}i+q_{j}j+q_{k}k,$$ (4)

where the quantities *q*_*r*_, *q*_*i*_, *q*_*j*_, and *q*_*j*_, are real numbers and *i*, *j*, *k* are unit-vectors pointing along the three spatial axes in the map frame. The (*X*, *Y*) data, used as the center for the obstacle position (i.e., the hole), are then sent to the function that uses ROS point cloud library (PCL) to create a virtual 3D cylinder. Once this virtual object has been created, it is then published by the ROS topic as it was acquired by the vision sensor of the smart wheelchair. The information about the created virtual obstacle is used for two purposes by two different packages: one package visualizes the point cloud creating a virtual object in Rviz, while the other, the ROS navigation stack, receives the virtual obstacle information through point-cloud sensor data and uses this to update the map in order to correct the navigation.

While the navigation task is realized, all the acquired data, the coordinates of the robot and the obtained holes coordinates in the global reference system, are elaborated. When a trigger (e.g., from the keyboard) is received, the coordinates of the holes are estimated as in Eqs 1, 2. As will be later shown in Section 3.1, the estimated positions can be compared with the real coordinates of the holes located on the map in order to evaluate the accuracy of the estimation. Then, a 3D cylinder is created, converted in a point cloud representation, and placed on the estimated position within the virtual map. The point cloud conversion is required in order to present the data as ROS sensor data, which is the format accepted by the navigation stack (the virtual obstacle is seen as if it were detected by an RGB-D camera sensor). The approach of using a point cloud image of the virtual object provides a fast representation, allowing a temporary modification both of the map and of the path planning of the mobile robot, which is also useful when there are different robots that operate in the same environment and that can share this information.

The robot path planning is executed in the ROS by the package called ROS navigation stack. It receives information from odometry and sensors to process this data and then executes commands on mobile base. As a pre-requisite for the navigation stack use, the mobile robot should have an ROS tf transformation package transform tree in place and be able to publish sensor data using the correct ROS message types. The navigation stack needs to be configured for the dynamics of the single mobile robot as reported in Figure 4. Before the robot starts to move, the global planner creates a path from the current robot position to the destination point by using a static map. Then, the local planner tries to match the global path with the provided robot kinematics information and all the available sensor readings in order to achieve the goal.

### 2.4 BCI protocol

The designed protocol aims to evoke ERPs defined as cognitive responses, elicited when the user reacts to unexpected sensory stimuli. The ERPs, detected mainly in the central region of the scalp, present a latency time of appearance (delay after stimulus presentation) that can vary accordingly to the complexity of the cognitive task, performed by the individual. In literature Fu et al. (2019), the ERPs were also used to investigate the neural correlates of deception when the participants completed a hazard judgment task, while in Qin and Han, 2009, these EEG potentials were recorded from human adults while they identified risky and safe environmental events. The ERP elicited after a user’s recognition of a task error is called error-related potential (ErrP) (Tang et al., 2021). The ErrP is generated when a subject commits or observes an error (Rousseau et al., 2012), and this aspect has interested many authors for its integration in a control loop for BCI systems. In literature, the ErrP can be defined by two main components: the first one is the error-related negativity (ERN or Ne), which is a negative potential peaking 0–200 ms after an erroneous response, and a second component, an error-related positive potential, called error-related positivity (Pe), that may follow the ERN depending on the task realized, with a latency of 200–500 ms after the error (Koban et al., 2010). Previous studies showed that ERN and Pe are specifically linked to error monitoring. In particular, the ERN is a defined event-related potential that is associated with performance monitoring at the response processing stage of goal-directed behavior, indexing early error monitoring (Overmeyer et al., 2021). Error-related negativity is triggered when a user either makes a mistake or the application behaves differently from the expectation. It can also appear while observing another user making a mistake (Vi et al., 2014).

The proposed protocol is designed based on the “oddball paradigm” (Wolpaw and Wolpaw, 2012) with the aim of evoking ERPs, connected with error performance activities and presented as different visual stimuli with the presence or not of obstacles on the robot path planning. In this paradigm, a sequence of events, which can be classified into two categories, is shown to the subject. One of the categories presents events that can be considered “target stimuli” and should have a lower frequency of occurrence with respect to the other. So, the overall task is consisting of 200 trials of short videos of robot navigation in indoor environments in presence of obstacles. The 80% of these trials show the robot smoothly navigating (i.e., no problem with obstacle occurrence), successfully avoiding a correctly detected obstacle by turning left or right. The remaining 20% of the trials represents when the robot collides with an obstacle (e.g., a small hole on the floor). The 200 trials are divided into four groups, each group contains 50 trials presented in a video of 7 min, for a maximum session duration of 30 min, including pauses presented among the event group presentation. The user, while seeing the video stream, is asked to press a specific key on the keyboard whenever he/she recognizes that the robot is going to pass through the hole or not. The scenario represents a mobile robot navigating autonomously in a simulation environment with a first-person camera view (FPV), like the person is sitting on a smart wheelchair. The obstacle (i.e., holes in the floor) appearing in the scene can be detected or not within 2 m by the sensory equipment from the smart wheelchair (Ehrlich and Cheng, 2018; Zhou et al., 2021). In each video clip, there is a directional cue, a simple arrow pointing left or right, as a prediction of the next mobile robot direction as presented in Figure 2. At this point, the simulated wheelchair may respect the directional cue, turning left (or right), thus avoiding the hole (correct trial, “Turn”), or it may go straight, ignoring directional cues and passing over the hole (non-correct trial, “Pass”).

**FIGURE 5:**
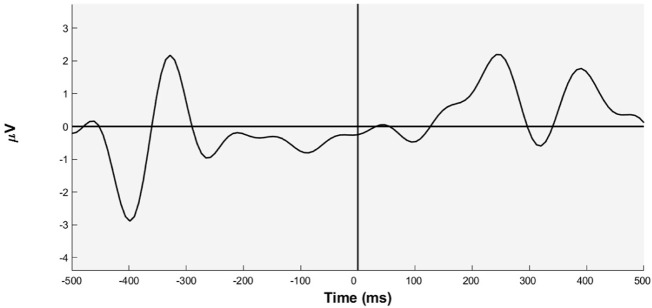
EEG signal averaged over the Fz channel when the robot is approaching the hole that occurs 40 times, that is the 20% target trials of all those proposed.

All recorded data and events are time-locked around an event marker synchronization, namely, “Time 0”, which is the moment the subject has pressed the key. Then, the signals have been epoched around this marker in a time window of 2 seconds, before and after the marker. In Figure 5 an evoked signal has been reported during the training phase, and while the robot is approaching the hole and passing through, the evoked potential has been obtained by filtering the epoched signal by keeping only theta-band through the band-pass filter in the range 3–8 Hz.

### 2.5 Brain computer interface system and EEG signal pre-processing

The study population is composed of 10 healthy subjects whose mean age is 28 years (standard deviation (STD) ± 3 years). The used laptop is HP Pavilion with 16 Gb ram and 10 G CPU, for recording the EEG brain signals. The BCI2000 software with G. Tec g. MobilabPlus DAQ and an actiCAP system have been used for the EEG signal acquisition Sawangjai et al. (2020). Eight channels were placed on the subject’s scalp, and the electrodes were arranged according to the international 10/20 system and placed at Fz Cz Po7 P3 Pz P4 Po8 AF8. The data were recorded with a sampling rate of 256 Hz, and band-pass filtering is applied between 0.5 and 30 Hz. Afterward, EEG data have been filtered again by a bandpass forward–backward filtering between 1 and 10 Hz (Butterworth second-order filter) was applied followed by decimation of a factor 8, introducing downsampling from 256 Hz to 32 Hz. The signals are further filtered in different bandwidths, and the best bandwidth in terms of classification accuracy, 2–5 Hz, is selected for ERP detection (Spüler and Niethammer, 2015). A Bayesian linear discriminant analysis (BLDA)–based classifier is considered to predict two groups, i.e., “Turn” or “Pass”. Among the proposed classifiers for ERP detection in the literature, BLDA was chosen since it is efficient and fully automatic (i.e., no hyperparameters to adjust), and due to its regularization, it can avoid the problem of overfitting of high-dimensional data or noise interference (MacKay, 1992; Hoffmann et al., 2008). The data collected from each subject have been divided into 60% for training and 40% for testing.

The implemented algorithm to identify the target task (when the robot does not detect present obstacles) works on time windows of 1 s during which the EEG potentials could be detected. As soon as the interested target event of committing an error is identified by the classifier, it will rise a flag to MATLAB Simulink, which will send a trigger to the ROS node.

## 3 Experimental results

As detailed in Sections 1 and 2, the robot navigation stack has been modified to receive the feedback from the user through an input device (e.g., keyboard and BCI). When the trigger is received, a virtual obstacle is generated and placed on the virtual map of the robot, centered on the coordinates estimated *via* Eqs 1–4. The effect of the virtual obstacle is that of affecting the local trajectory planner of the robot, which avoids the real obstacle as long as its position is close to the estimated one.

In Section 3.1, we present the results obtained for estimating the obstacles’ positions in a simulation environment, when the trigger is generated by pressing a key on a keyboard. The aim of the experiments is to validate the proposed human-in-the-loop approach, while providing the baseline performances, which can be obtained by means of simple but effective input devices. Section 3.2, instead, reports the results obtained by using the BCI protocol as of Section 2.4 in terms of classification of the ERP, where the error event is represented by an obstacle along the robot path, which is observed by the user but not detected by the robot sensory set.

### 3.1 Robot navigation simulation results

The data collected during the proposed robot navigation trials are those described in the tasks 1) and 2) presented in Section 2.1.

The users sit in front of the screen, and they see the robot simulation during real-time navigation, as illustrated in Figure 2. Users have to press a keyboard key in case they see an obstacle centered on the screen.

#### 3.1.1 Results of task 1: Navigation between the obstacles

**FIGURE 6:**
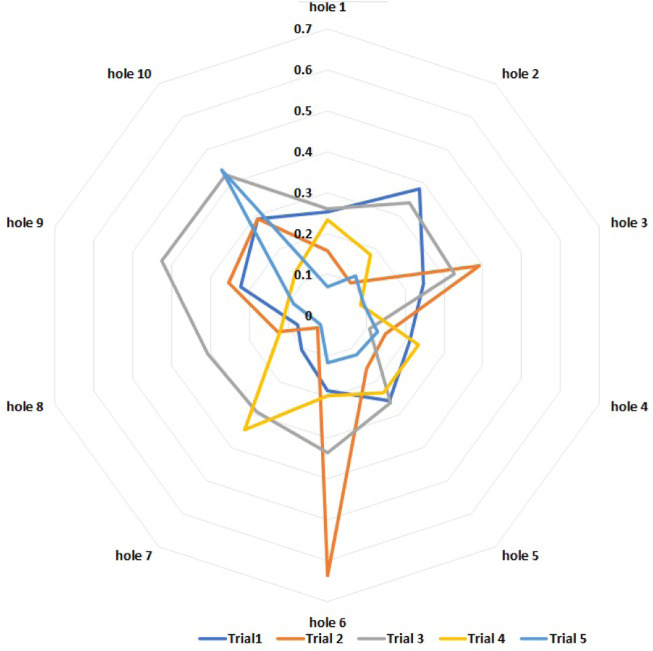
Task 1: estimation errors of the positions of each hole during five trials (one color per trial, errors in meters).

Figure 6 shows the results related to the five trials of the first task. In particular, it shows the distribution of the estimation errors of the obstacles’ positions, i.e., real position minus estimated position. The estimated position is calculated by Eqs 1–4 when the user generates the trigger with the keyboard. In Table 1, the main statistical indexes related to the estimation errors are reported. It should be noted that in robotics, obstacle avoidance is commonly performed by considering the obstacle area increased by a safety threshold, namely, the inflation radius, which depends on the robot in use. If the error between the estimated position of the obstacle and the real one is less than the inflation radius, then the center of the virtual obstacle will fall within the inflated area of the real one and vice versa. As such, the inflation radius is considered the maximum acceptable error. In the specific case of the wheelchair in use, the inflation radius is set to 0.5 m.

**TABLE 1:** Task 1: Statistical indexes of the estimation errors in the positions of the obstacles.

Error	Value	Unit
Average	0.21	m
Minimum	0.03	m
Maximum	0.64	m
Standard deviation	0.12	m
Variance	0.02	m^2^

#### 3.1.2 Results of task 2: Navigation through the obstacles

**FIGURE 7:**
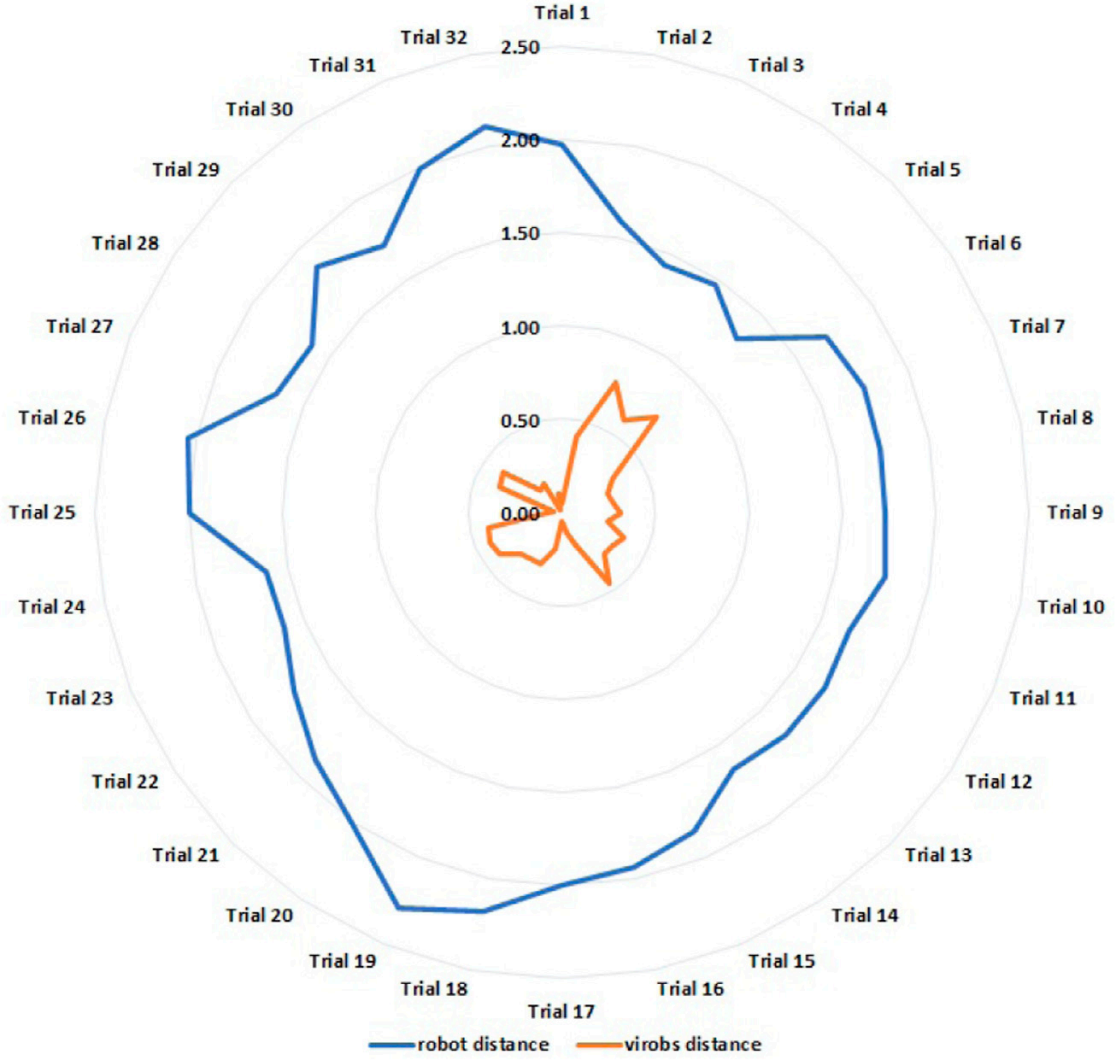
Task 2: estimation errors of the position of a single hole (orange) and relative robot distances (blue) during 32 trials (values in meters).

Different from the previous case, this time the wheelchair randomly crosses a single obstacle from 32 different starting points. Depending on the approaching direction, the trigger from the user can be generated before or after the hole is actually shown at the center of the screen (Figure 2). As a consequence, the virtual obstacle can be centered before or after the real one. In order to preserve the sign of the estimation error, Figure 7 now shows both the error and the relative robot distance for each trial, namely, the distance between the robot and the obstacle at the moment in which the trigger is generated. Table 2 presents the main statistical indexes related to the estimation errors.

**TABLE 2:** Task 2: Statistical indexes of the estimation errors in the positions of the obstacles.

Error	Value	Unit
Average	0.30	m
Minimum	0.02	m
Maximum	0.75	m
Standard deviation	0.178	m
Variance	0.02	m^2^

Resuming the presented data, it is possible to notice that in Table 1, referring to the first proposed task, the maximum error reported is 0.64 m, while in Table 2, the maximum error estimated is of 0.75 m. The two values are both greater than the accepted error threshold of 0.50 m. Moreover, the average error is 0.21 m for the first task and 0.30 for the second one: both are small and represent an estimation accuracy which falls within the threshold of the inflation radius. The minimum error reported in the two tables is of 0.03 m in Table 1 and of 0.02 m in Table 2. The precision can be evaluated by considering the standard deviations, namely, 0.12 m for the first and 0.178 m for the second. This aspect can be expected because any time the robot does not realize the correct path planning, it can move randomly around the obstacle as can be observed in Figures 6, 7.

### 3.2 ERP classification results

The results presented in this subsection are related to the ERP detection for the human-in-the-loop control feedback, namely, task 3) as of Section 2.1. The BLDA classifier is considered for binary classification because features are classified into two groups (“Turn” and “Pass”). The area under the curve (AUC) of the receiver operating characteristic (ROC) curve is considered to evaluate the performance of ERP detection (Dubitzky et al., 2013). Good AUC values range from 0.5 to 1; the latter meaning perfect classification of elements belonging to both positive and negative classes. Table 3 shows the results of offline ERP detection. The results are obtained considering a time window of 1 s (i.e., 1 s from “Time 0” and all channels for classification). The EEG signals are filtered considering the bandwidth of 2–5 Hz. The bandwidth 2–5 Hz has been exploited for classification due to its best performance in terms of AUC.

**TABLE 3:** AUC index of ERP detection obtained from 10 healthy subjects.

Subject	AUCtrain	AUCtest
S001	0.85	0.61
S002	0.96	0.72
S003	0.73	0.53
S004	0.63	0.51
S005	0.64	0.54
S006	0.61	0.55
S007	0.73	0.66
S008	0.73	0.62
S009	0.69	0.53
S010	0.55	0.45
Mean	0.73	0.59
Standard deviation	0.11	0.07

**FIGURE 8:**
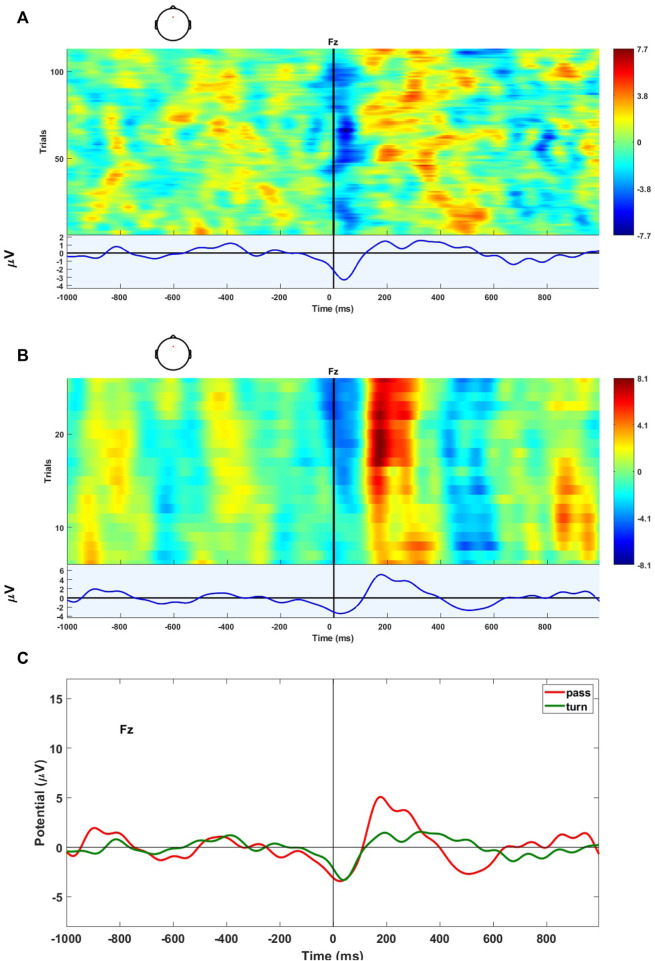
ERPs of subject S002. **(A)** Fz channel for the “Turn” group (i.e., the robot successfully avoid the obstacles). **(B)** Fz channel for the “Pass” group (i.e., the robot failed to avoid the obstacles). **(C)** Grand average over the Fz channel for two conditions, “Pass” and “Turn”.

Subject 2 shows the best result in terms of AUC both for training and testing. Figures 8A,B show the ERPs of subject S002 over the Fz electrode for “Turn” and “Pass” groups. Each line along the *y-*axis is related to one trial, while on the *x-*axis, the single time instants are represented. The black line denotes the time the trigger is received by the user to synchronize the signals. Finally, in the bottom part of the figures, the grand average ERP has been shown.

In Figure 8C, the grand average ERPs have been analyzed to highlight the differences between the two different conditions “Pass” and “Turn”. The figure reveals the grand average morphology that has the appearance of an error-related potential when the user recognizes that the robot is going to pass through the hole (non-correcting trial).

## 4 Conclusion and discussion

The proposed study has presented a method to integrate the navigation realized by a mobile robot, equipped with onboard sensors, and a human feedback to deal with possible obstacles that are not correctly detected by the mobile robot. The feedback is provided by both a keyboard and a brain–computer interface, and for this purpose, the work includes also a protocol design phase to elicit ERP potentials. Moreover, the proposed approach can be suitable for any mobile robot that navigates indoor or in ambient assisted living (AAL) scenarios, trying to overcome all the issues related to the erroneous sensor reading in an indoor environment (Silva et al., 2018; Cojocaru et al., 2021).

The proposed architecture has been first tested using a keyboard to generate the feedback in order to validate the baseline performance, showing suitable results for the aim of involving the user in the control loop. The obtained results support the strategy of the proposed approach of using virtual obstacles for mobile robot trajectory correction through visible and applicable point cloud objects, giving good accuracy and precision about the exact negative obstacle’s position (i.e., holes in the ground). Among the advantages of this approach, there is a reduced computational request that is advantageous for a real-time application.

The human feedback can be generated with any common input device, as well as with a BCI. In detail, a protocol to elicit the ERP signals has been proposed. The ERPs elicited after the user recognizes a task error are defined as ErrPs. Through a BLDA classifier, considered for the binary classification, it has been possible to classify the evoked signals into two groups (“Turn” and “Pass”). The obtained results have highlighted the differences between the two conditions with a signal morphology obtained by the signals’ grand average that presents the appearance of an error-related potential, particularly when the mobile robot is approaching the hole going through it.

The method is independent of using a specific mobile robot, but the accuracy can vary with respect to robot speed and the positions of the obstacles. In the proposed work, the realized case study plans the wheelchair top speed as relatively low for people with special needs, and the mobile robot has set a value of 0.5 m inflation radius in the navigation stack. In case of motion speeds greater than 1 m/s, the algorithm should consider a compensation in order to account for possible delays between the moment in which the obstacle is seen by the user and that in which the trigger is received by the navigation stack. A limitation of this concept is the obstacle estimation, which occurs in a fixed position of 2 m in front of the wheelchair, so it only informs the robot if there is an obstacle or not within this distance. Moreover, by default, it creates a virtual obstacle of fixed size, regardless of the size of the detected real obstacle, and this will require further improvements in the future to detect and provide the robot with information of different sizes and distances. Some disadvantages of the proposed approach are related to the detection of ERP, which is not quite good for some subjects; then, for them, it is not possible to perfectly detect all obstacles. Moreover, the training of the data analysis algorithm is time-demanding and is subject-dependent. The proposed BCI protocol is synchronous; thus, a possible future research direction could be the investigation and the introduction of asynchronous protocols in the framework in order to test more realistic scenarios.

## Data Availability

The raw data supporting the conclusion of this article will be made available by the authors, without undue reservation.
